# Preventing tobacco in vocational high schools: study protocol for a randomized controlled trial of P2P, a peer to peer and theory planned behavior-based program

**DOI:** 10.1186/s12889-018-5226-y

**Published:** 2018-04-13

**Authors:** Florence Cousson-Gélie, Olivier Lareyre, Maryline Margueritte, Julie Paillart, Marie-Eve Huteau, Kela Djoufelkit, Bruno Pereira, Anne Stoebner

**Affiliations:** 1 Univ Paul Valéry Montpellier 3, Univ. Montpellier, EPSYLON, EA 4556, F34000, Montpellier, France; 2 Epidaure Prevention Department of the Regional Institute of Cancer of Montpellier-Val d’Aurelle, Montpellier, France; 3 CHU Clermont-Ferrand, Biostatistics Unit, Délégation de la Recherche Clinique et des Innovations, Clermont-Ferrand, France

**Keywords:** Tobacco prevention, Peer-to-peer, Theory of planned behavior, Young people, Vocational high school

## Abstract

**Background:**

In France, the issue of youth smoking remains a major challenge for public health. School failure, socio-economic and socio-cultural backgrounds influence the initiation and maintenance of smoking behavior in adolescents. Vocational students are at particularly high risk of using psychoactive substances, including tobacco. One of the most important factors is the environment, whether family, friends or peers. Therefore, peer education has a positive potential to change smoking behavior of adolescents. It has also been demonstrated that the Theory of Planned Behavior (TPB) has yielded the best prediction of intentions and behavior, in several health domains, including on tobacco. However, it is usually confined to the measurement of processes by which interventions change behavior, rather than to the development of these interventions. The objective of this paper is to describe the protocol for a randomized controlled trial of a peer intervention based on the TPB on a highly exposed young population.

**Methods/designs:**

This is a cluster randomized controlled trial comparing an intervention group to a control group, randomized into clusters (professional schools and classes) and stratified in three departments (Hérault, Aude and Gard) in the Languedoc-Roussillon region. The primary issue is the prevalence of daily smoking at 24 months, defined by a daily tobacco use of at least 1 cigarette, validated by CO levels in exhaled air. The primary hypothesis is that intervention will lead to decrease the daily smoking prevalence of 10% between the intervention group and the control group during a 2-year follow-up.

**Discussion:**

The results from this trial will provide evidence on the effectiveness of an innovative peer-to-peer intervention based on the TPB.

**Trial registration:**

ISRCTN: 37336035, Retrospectively registered 11/12/2015.

## Background

Risky behaviors, especially smoking are the major causes of morbidity and mortality. In France, the issue of youth smoking remains a major challenge for public health: The initiation age to the first cigarette and an increase in smoking among teenagers, especially between 13 and 18 years old, smoking prevalence goes from 5% at 13 years old to around 38% at 18 years old [1]. This prevalence is even higher in teenagers attending vocational schools [2–7]. It has even reached 43% in the Languedoc-Roussillon region [7].

Smoking initiation factors are multiple [8]. One of the most important is the environment, whether it is family, friends or peers [9]. Other factors such as school failure, socio-economic and socio-cultural background, influence the initiation and maintenance of smoking behavior in adolescents. For instance, at the age of 17, the daily smoking was 21.4% for students who have never repeated a year in school, 38% for those who have repeated it once and 42.1% for those who have repeated it twice or more. Thus, multiplying the risk of daily smoking by two [10]. A survey carried out by the Academy of Montpellier shows that vocational students have repeated school years more often because they received their High-School Degree at a later age than the other students [11]. Therefore, vocational students are particularly at high risk for using psychoactive substances, including tobacco. In addition, in adolescence, the temptation of experimenting and becoming a daily smoker is an important risk factor for sustainable consumption and dependence [1]. Smoking is a learned behavior, an “allowed” and supported learning to varying degrees by the culture that surrounds the individual: A learning that leads to dependence [12].

The risk is even greater since most of these adolescents are strongly influenced by their friends and their perception of what is acceptable or not in a group regarding smoking. Peer influence in the initiation of tobacco use has been extensively studied. Having smokers among one’s friends is one of the most powerful and most consistently identified predictors of teen smoking [12–16]. Peer influence is normative, but not injunctive. Whereas, adolescents would actually take a direct pressure to smoking as an aggression and would break friendship bonds, young people feel an internal pressure and start smoking not only from fear of losing contact with smoking friends, but also to achieve a sense of autonomy and independence.

In addition, adolescents tend to overestimate the prevalence of smoking among their peers, up to 50% more [17]. This misperception is likely to be a determinant of smoking, which is more important than the actual use of tobacco by friends [18]. This normative effect is then reinforced by the relational change that occurs, causing the smoker to approach new people with a similar behavior [14]. If peer influence is normative and can encourage tobacco use, we observe that the influence against tobacco is also present [19]. Therefore, peer education has a positive potential to change the smoking behavior of adolescents.

In the past, educational interventions to control tobacco use among young people were mostly based on the *information deficit model* or on the *rational model*, in other words the priority was to provide information on health risks and negative consequences of smoking. This model has certainly been considered to be a crucial and necessary step, but has not been sufficient in changing the subsequent behavior [12, 20, 21]. The second type of intervention was based on the *educational emotional model* or *social skills curriculum,* which showed a low or negligible impact on the behavior [17, 22, 23].

Today, tobacco prevention is based in part on *the model of social influences*, where the emphasis is on the youth’s social environment, namely resistance to social influences and normative education [17, 24]. Young people have resources, dynamism and creativity. They speak the same language and are able to build strong trusting relationships with peers who understand their life circumstances and with whom they can communicate their ideas in a simpler way, that is to say in an understandable language. It is the mark of peer education. The same information can be delivered in different ways depending on the peers and the public [25].

Adolescents reckon that dealing with people their own age is positive [26, 27]. They also have the impression that peers are more likely to understand them. They are confident and can then say what they really think. Peer education could allow teenagers to appropriate intervention and strengthen their capacity to act. One study even showed that peer education could promote youth engagement in a sustainable dynamic of their own and allow them to be part of community development solutions rather than being submissive or passive [28]. With the support of the community and specific training, teaching peers can be powerful allies in prevention efforts.

Resorting to the peer-to-peer (P2P) approach could be an effective way to prevent risky behavior since young people are more likely to listen to those who resemble them. Various studies have shown the positive impact exerted from youth education by teaching peers [12], but the impact level of this strategy on peer behavior has long lacked solid evidence to affirm the long-term effects, as well as the relevance of such interventions in high schools [29–31]. Over the past ten years, peer education has been used in various fields. In Albania, a campaign of peer education has been carried out to sensitize teenagers to HIV and condom sales have rapidly grown [25]. In Australia, a song was created to inform young people about drug addiction, which resulted in an increased participation of street children [25]. The model “Natural Helpers” aims to recruit natural caregivers to train them and to bring them to teach. This model has been successfully used as a strategy in the prevention of drug addiction, violence and suicide [25].

Peer education has also been used in tobacco prevention. The project « SURICATES » [32] in France, has shown that students were better informed about cigarettes thanks to a peer education campaign. This study also showed differences in opinions, tobacco depictions, and perception of risk, but these findings were not statistically significant. In 2001, the peer method was used in Greece to produce a visual support providing an anti-smoking message [33]. The study showed a significant increase in anti-tobacco attitudes after the intervention up to three months later. A significantly increased knowledge of addiction and tobacco consequences was also reported. More recently in 2009, the project “MYTRI” conducted in India was successful in reducing teenage smoking [34]. As for the “ASSIST” program, it spotted young “leaders” in high schools and trained them to act as teaching peers to sensitize youth to tobacco [35]. Even if the interrogated peers thought they strengthened non-smoking behavior more than they helped regular smokers to stop [36], the prevalence of smoking among adolescents was efficiently reduced [37, 38]. Peer-to-peer interventions seem to be a promising method for student tobacco prevention, from the development of a program to its implementation.

Yet, if adolescents are best placed to predict their friends’ needs and to be listened to by them, a framework must be anticipated and maintained [39]. Indeed, a scientifically validated theoretical basis must be ensured and method relevance must be verified as recommended by experts in behavior change [13–40].

Researchers can now rely on a number of behavior explanatory models to develop their interventions. One of the most relevant theories in smoking prevention is the Theory of Planned Behavior (TPB) [41]. It postulates that a person’s behavior is largely determined by the person’s conduct control perception and intention to adopt it. This intention would in turn be explained by attitude, subjective norm and perceived behavioral control of the person.

**Fig. 1:**
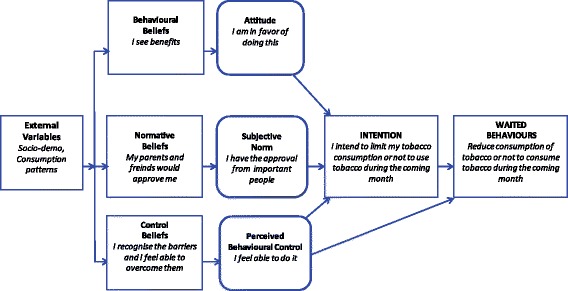
Model of theory of planned behavior

Attitude, guided by behavioral beliefs (subjective probability that a behavior will lead to a particular instrumental or affective outcome and the importance of such an issue), indicates a more or less likely adoption of a specific behavior. Subjective norm, determined by normative beliefs (perceived expectations or behavior of significant others, combined with the motivation to comply with this entourage), is the perceived pressure applied by important people or groups of people toward behavior approval. Finally, perceived behavioral control, following control beliefs (perceptions of probability of occurrence of factors facilitating or hindering behavior adoption and their respective weight) is defined as the perception of the degree of ease or difficulty by which a giving behavior can be adopted (see Fig. 1).

The TBP has been used to study various behaviors (diet, physical activity, health behaviors, screening, condom use, ecological behavior, etc.). Being compared many times to other models, the TPB has yielded the best prediction of intention and behavior [42, 43]. Overall, the TPB model explains 35–55% of the intention variance and 25–35% of the behavior variance [41, 44–52]. In the meta-analysis of Armitage & Conner [45], which includes 161 studies and integrates all types of behavior, intention was explained, in order of importance, by attitude (*R*^*2*^ = .24), perceived behavioral control (*R*^*2*^ = .18) and subjective norm (*R*^*2*^ = .12). Behavior is explained by intention (*R*^*2*^ = .22) and perceived behavioral control (*R*^*2*^ = .13). Nevertheless, it is interesting to note that the relationship between social norm and behavior is higher among adolescents than among older populations [53].

In 2010, Topa and Moriano published a meta-analysis based on structural equation modeling of TPB and tobacco [51]. Their results indicate a good model fit, with attitude, social norm and perceived behavioral control explaining 16%, 20% and 24% of the intention variance, respectively, the latter explaining in turn 30% of behavior variance. However, the authors did not distinguish between the intention to smoke, to cut down or to quit. A 2005 literature review, on previous articles, did not reveal major differences between these three types of intentions, 34% of the variance of each being explained by the model [54]. On a teenage audience, the TPB has also demonstrated its effectiveness in predicting smoking, both in terms of intention and behavior [20, 55–58].

If the TPB is now supported by significant studies justifying its theoretical relevance, many authors still deplore its application. Indeed, it is confined to the measurement of processes by which interventions change behavior, rather than to the development of these interventions [59]. Thus, the TPB is rarely cited as an underlying theoretical basis and when some of its components are identified, it generally remains at the sole interpretation of the reader. One or two elements may be detached from the whole TPB model and often associated with elements relatable to other theoretical models, or to no model at all. More generally, interventional research articles explain little about the content and the place of interventions, which makes them hardly comparable, reproducible and analyzable [40]. The results of these intervention studies should therefore be interpreted cautiously.

The literature review of Hardeman et al. only identified 24 programs that explicitly mentioned TPB, and sometimes mixed with other theories [60]. Only two of them studied tobacco consumption, and the TPB model was used only as a measuring tool. As for the other types of behavior, the journal mentions 12 interventions based at best on some components of the TPB, and concludes that intention changes are low and behavior changes are low to moderate. Since 2002, other interventions have been based on the TPB and have resulted in similar effect sizes [61–64]. Nevertheless, it appears that interventions based on the TPB produce significantly higher effects than those based on the *transtheoretical model* or the *social cognitive theory* [61].

Conducting a program on vocational students to prevent smoking behavior remains a major public health issue. Therefore, proposing to vocational students to develop and implement this intervention with their peers, based on the TPB model, represents a promising and necessary innovation.

## Methods/design

### Study design

This is a cluster randomized controlled trial. The intervention group will be compared to the control group after randomization into clusters (professional schools and classes) stratified in three departments of France (*Hérault*, *Aude* and *Gard*). The intervention will be a smoking prevention program made by the peers, based on the Theory of Planned Behavior (TPB).

### Recruitment

The P2P project will be developed in the departments of Aude, Hérault and Gard. The departments of Pyrénées, Orientales and Lozère will not participate in this project for two reasons: either with regard to the proximity of Spain, which is a country where the price of tobacco is lower than in France, or with regard to the lack of vocational high schools. Indeed, only vocational high school students will participate in this research and in the department of Lozère, there is only one, which may falsify the randomization. The project team will meet with the head teachers, the Principal Educational Adviser and the school nurses from 20 vocational high schools of the three departments.

### Participants

The study includes students, both girls and boys, in Year 11 of vocational school in three departments of France. All of the participants are required to be able to speak and read French. Exclusion criteria are: not wanting to attend the evaluation, not able to speak or read French, and not able to have a follow- up (e.g. due to change of schools in the two years).

### Ethical approval

Favorable ethical approval for the trial was given by the French ethic comity "Comité de Protection des Personnes (CPP) Sud Méditerranée I" on July 24, 2013.

### Informed consent

Prior to being visited by a research assistant, each student who is invited to participate in the clinical trial will be given an information sheet by the research team. There will also be information for parents about our request to involve their children in the trial.

Informed consent will be obtained from all participants prior to the trial. For those under the age of 18 years old, who will be included in the research trial, at least one parent will have to give consent for participation. Those over 18 years old will be able to give consent without parental approval.

### Randomization

The trial will be randomized into clusters (vocational high school) and stratified in three departments of France (Hérault, Aude and the Gard). As the study will be carried out throughout several departments where smoking consumption levels and socio-demographic parameters differ, the randomization will be stratified by department. The risk of interpenetration (or contamination) between schools will be minimized by retaining vocational high schools from different geographic areas. The classes from the same school will not included in two different randomization groups.

Randomization by random blocks will be performed using Stata software (version 13, StataCorp, College Station, US) by an independent statistician and considering stratification according to area of the region (department) and size of school (in terms of number of children per school).

### Intervention

The intervention will be a peer education process based on the Theory of Planned Behavior (TPB). Within interventional schools, the community program will be designed by voluntary stakeholders of the *Maison des Lycéens* (MDL, a student association in every high school, controlled by the institutional authorities) following a predefined framework. Every TPB dimension will be developed by these peer educators in action for smokers (casual and / or dependent) or non-smokers. A “blank” action matrix will be initially proposed to students and will allow the distribution of the interventions suggested by the young people in corresponding boxes. The content will be tailored to each of the community-school needs.

A technical committee will accompany the students teaching peers to help them achieve their objectives. The committee’s aim will be to support creativity in each interventional MDL and to bring supplementary elements if needed and according to their wishes. The proposed interventions will take into account all fundamental factors known to promote youth participation in action and should avoid the known obstacles that usually lessen their involvement [65, 66].

Thus, all actions will incorporate the following elements: free, confidential, accessible, based on volunteering and empathy. The activities will allow the active participation of young people in groups and will be enjoyable with a number of 5 ≥ sessions in case of a weaning group. There will be support from friends, peers, teachers and parents, taking into account addiction. The project team will ensure that every high school student who wishes to quit smoking may have access to a quitting assistance if desired.

Six guidance sessions, each lasting a maximum of 1 h, will be proposed by the technical committee to the peer educators of the *Maison des Lycéens* (MDL) in the intervention group. The defined content for these sessions is follows:

Session 1: presentation of the teams, aim and protocol for the study; constitution of the group of voluntary peer educators; if necessary, recommendations to enable young elected representatives of the MDL to recruit other peer educators amongst the non-elected high school students (friends, leaders…).

Session 2, 3 and 4: gathering of intervention propositions thought up by the youth of the MDL, verification of the conformity between the interventions proposed by the youth and the TPB; orienting them, if necessary, in order for the interventions to cover every dimension of the TPB, and, in last resort, propositions of “turnkey” examples of actions; provisional budget estimate per action, adjustment of the interventions/budget if necessary, and final validation of future interventions.

Session 5 and 6: provision of the necessary (material, financial) means; contribution of knowledge and expertise in order to elaborate the tools if necessary; support in the elaboration of the tools; validation of the intervention tools.

The actions proposed by the peer educators, with the support of the technical committee, will be carried out during the remaining months of the school year. The pace of the interventions will be tailored to meet the requirements of each high school according to the needs and modes of organization. The length of each action will be adapted to the time realities of each school: either on a duration going from one to two hours, or one day dedicated to the actions. At the end of each school year, a day of meetings and exchanges will be organized between the high school students of the MDL, part of the intervention group. This day will take place at the Epidaure Centre and will gather youth from the MDL, the local education authority, the Languedoc-Roussillon Regional Council and the *ARS* (Regional Health Agency) representatives, as well as Public Health professionals and teams from the educational community.

The goals of this day for the MDL youth will be to meet other young elected representatives of the MDL from other intervention schools, to exchange on their own practical experiences, to learn about other proposed intervention tools and to present their own created intervention tools, as well as to discuss health, health education and nicotine related addiction with specialists and health psychology professionals and to evaluate the knowledge and expertise acquired during the year.

The aim for the professionals will be to evaluate the impact of the actions on the peer educators, to discover all of the tools created and to discuss with the MDL youth.

### Objectives

The primary objective of this study is to measure the 24-month impact of a peer intervention based on the TPB on a highly exposed young population (from Languedoc-Roussillon vocational schools) by reaching a 10% difference in daily smoking prevalence between interventional and control groups after two-years.

Secondary objectives are to increase intention to not start smoking and/or intention to diminish tobacco consumption. This study aims to modify norms in favor of a tobacco non-consumption: behavioral norms (“I see benefits”), normative beliefs (“my parents and friend(s) would approve”), control beliefs (“I recognize the difficulties and I feel able to overcome them”), attitudes (“I am in favor to do so”), subjective norms (“I am approved by people that are important to me”) and perceived control (“I feel capable of doing this”).

### Outcome measures

The **primary outcome measure** is the prevalence of daily smoking at 24 months defined by daily tobacco use of at least 1 cigarette validated by CO levels in exhaled air.

The **secondary outcome measures** are: using habits (tobacco, alcohol, cannabis); tobacco consumption modification characteristics (decrease, quitting); tobacco addiction and CAST score, elements of TPB (behavioral norms, normative beliefs, control beliefs, attitudes, subjective norms, and perceived control); gender; environmental quality of high schools toward tobacco.

### Assessments and data collection

#### Baseline

An online evaluation questionnaire will be proposed to all students from the control group and from the intervention group to be filled during school class. It will address the following 5 themes: socio-demographic description of the individual; user habits (tobacco, alcohol, and cannabis), assessment of the level of dependence on tobacco and cannabis; motivation to reduce or quit smoking, elements of the TPB. Socio-demographic information includes gender, age, educational attainment of parents, indicators of precariousness (family ZIP code, perceived wealth and living conditions), family environment (place living, siblings) and school environment (boarding industry, repetition). Using habits (tobacco, alcohol, cannabis) and addictions issues were addressed by questions either internationally validated [67, 68] or used in surveys conducted in France [1, 69–71] for example, the “try” (at least once during lifetime), occasional use (<1cig/d), recent use (at least once during last 30 days), daily use, the time between waking up and the first cigarette craving. The TPB variables i.e. behavioural beliefs, normative belief, control beliefs, attitudes, subjective norms, perceived behavioural control and intention will be developed and measured under Ajzen’s recommendations [72].

#### Follow up

Students will be followed at 3, 9 and 15 months past baseline, using an online evaluation questionnaire and a CO-tester supervised by research assistants. The procedure will be filled out during a school class, for an hour long session, four times during the evaluation: before the beginning of interventions in December 2013 and January 2014, at the end of the first school year in April and May 2014, at school-start in October and November 2014 and finally in April and May 2015. The same measures, questionnaires and procedure of data collection will be used during the four evaluations.

#### Sample size

**Fig. 2:**
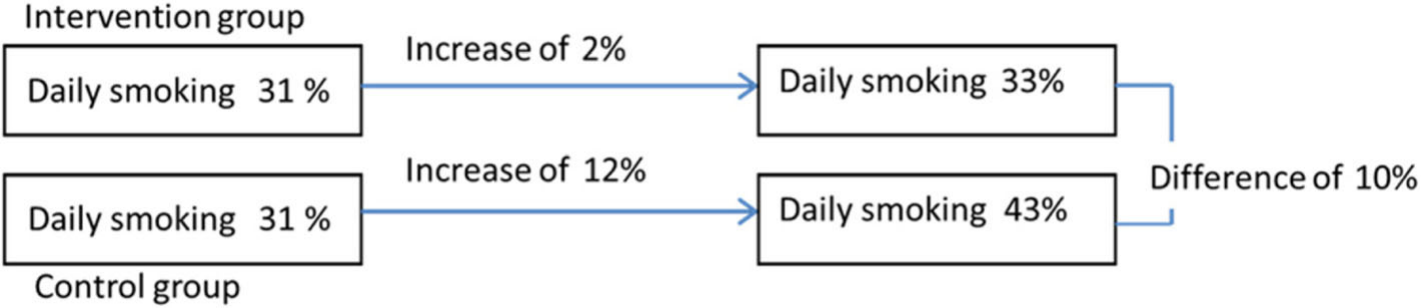
Expected 10% difference in daily smoking prevalence between interventional and control groups

The sample size estimation has been carried out on the basis of the literature [73, 74], with a daily smoking prevalence at 43% and a minimal absolute difference at two years between intervention and control groups equaling 10% (cf. Fig. 2). The two-sided type I error and the statistical power are fixed respectively at 5% and 80%.

To take into account the impact of cluster randomization, sample size will be multiplied by the variance inflation factor: 1 + (m(1 + cv^2^)-1)ρ with m the mean number of subjects per cluster, cv the coefficient of variation equal to ratio between the standard deviation of the cluster sizes and m, and ρ the intra-class correlation coefficient, which can be seen as the measure of the cluster effect.

If it seems appropriate to randomize the schools in order to avoid highly important contamination bias for the classes of one same school, it also seems essential to consider intra-class correlation, which is surely more important than intra-school correlation itself. In view of numerous studies where randomization is carried out according to this principle, ρ is fixed between 0.10 and 0.20.

Therefore, 740 participants are required according to the previous assumptions, without taking into account cluster effect. Considering mean of students per class around 20 (standard deviation of 4.9) and an ICC equaling 0.1, 2240 participants are required. Considering a lost to follow-up rate at two years around 25% and between and within school variability greater than previous assumptions, it seems reasonable to include 3000 participants for this study, which corresponds to around 125 classes and 14 schools.

### Statistical analysis

Analyses will be performed using Stata software. All data will be analyzed by intention-to-treat. The tests are two-sided, with a type I-error set at α = 0.05. Baseline characteristics (schools and children) will be presented as mean ± standard deviation (SD) or median [interquartile range] for continuous data and as number of patients and associated percentages for categorical parameters. Hierarchical generalized linear regression models (mixed models with logit link function for dichotomous dependant endpoint), with levels (random-effects) for schools, classes and children, and repeated measurements, will be used to estimate effect of the intervention on smoking prevalence. These models (intercept and slope as random effects for longitudinal analyses) include an interaction between randomization group and time-points evaluation, and will be adjusted on initial consumption (occasional smoking or daily smoking) and other epidemiological relevant parameters (duration and typology of smoking habits): age of first consumption, gender of children and school’s characteristics. Intra-class correlation coefficients will be presented by arm and results are described as odds-ratios and 95% confidence intervals.

The secondary analyses will compare changes between groups with random effect models (1) to measure the behaviors with regards to tobacco and cannabis consumption and their evolution over a period of two years and (2) to determine predictive factors of tobacco consumption.

The behaviors regarding smoking having theoretically been well differentiated in this population according to gender, a subgroup analysis will be explored as described previously.

To assess the problem caused by missing data (schools, classes and/or children), estimation methods developed by G. Verbeke and G. Molenberghs [75] will be proposed after sensitivity analyses particularly appropriate to measure the nature of missing data (missing at random or not).

### Process evaluation

As emphasized by Hawe, Shiell, Riley and Gold [76], an evaluation of the implementation of an intervention in health education context is essential. Indeed, the pre-existing context can influence the effectiveness of the intervention. The method suggested by these authors is based on a mixed methodological approach, both qualitative and quantitative. We will assess a P2P program with vastly used items such as the course of actions and the mobilization of participants (see items 1 and 2 of Table 1 below). As promoted by Hawe et al. [76], we will also analyze how the intervention was implemented in context (see item 3). To this end, we will run a network analysis between major actors of the program: members of the school board, members of the educational staff (teachers, directors), teaching peers, beneficiary peers, and members of the technical committee. This will be achieved both in the control group (networks within the home school and inside the school) and in the intervention group (network among peers and with the project members) at the beginning, central and final steps of the program.

**Table 1:** P2P process evaluation items

ITEMS	CRITERIA	INDICATORS	TOOLS
1. Did actions go according to plan?	• Compliance with provided protocol • Respect of schedule • Running in intervention and control groups	• Several steps • Step duration • Completion of each step • Measurement of deviation from the protocol	• Protocol drafting • Organization of indicators continuous reporting • Log book • Schedule
2. Did actions mobilize participants?	• Participation: - Project’s Technical Committee - Beneficiary peers - Teaching peers	• Regular attendance • Representativeness	• Attendance sheet
3. How did the actions take place in this context?	• Network analysis: - Members from the high school house of students - High-school teachers - Teaching peers - Beneficiary peers - Project’s technical committee	• Network density: number of contacts between members • Centralization: marginal or not based on the member’s role	• Individual meetings with key program actors • 3 evaluations (start, middle, and conclusion of the program)
4. How were the actions planned with the teaching peers?	• Issues and assets when taking actions • Teaching-peers needs • Relationship with technical committee	• Teaching peers needs analysis (relevance) • Adequacy of means to needs (consistency) • Review of actions	• Interviews with the teaching peers at the end of first year and second year of the program
5. How can the P2P program induce changes in high schools?	• Changes in the no smoking policy in high schools	• Changes in the high school students environment	• Number of changes • Time-scale change (short term, long term)

How actions took place with teaching peers is a crucial element for program success (see item 4). Thus, semi-structured interviews with teaching peers will be made to assess the issues and assets as well as the balance between the needs and the means at their disposal.

Finally, we will evaluate the events that might have occurred throughout the program such as the implementation of smoking cessation consultations in schools, the enforcement of no smoking rules in the facility, etc. (see item 5).

Qualitative data from the process evaluation will be subjected to a thematic content analysis. Key themes will be developed into an analytical grid. Each interview will be transcribed and coded into the grid themes. Quantitative data on fidelity and participation will be analyzed as covariate of outcome effects.

## Discussion

Smoking is a major challenge for public health and is associated with a range of factors. One of the most important factors is the environment, whether it is family, friends or peers. If the peer influence is normative and can encourage tobacco use, their influence against tobacco has been identified too. Prevention interventions, which promote these protective factors could have the potential to have a significant impact on the health and wellbeing of young people.

The P2P Program stemming from youth dynamism, its altruism and its new ways of communication [77] should boost the circulation of a validated piece of information into a risky population and its relatives, encouraging them to take into better account the major issues raised by smoking [39]. The very modernity of this program, with the original creation of a peer-based intervention mode that leads to a quality isometric confidence relationship between experts and public, should lead to the implementation of much appreciated interventions that fulfill the needs of the targeted population.

Prevention interventions need to be theoretically based during their development and to be evaluated with a rigorous methodology before expanding its implementation. This trial will assess the effectiveness of a prevention intervention based on the TPB. This theory has shown its effectiveness in predicting behavioral change and the not-starting-smoking-intent should reduce medium-term prevalence of smoking among the most exposed young people.

The results of this trial will help to inform future decision makers about the implementation of P2P in France.

If the P2P program shows an impact in the Languedoc-Roussillon region, larger-scale experimentation could reduce medium-term smoking prevalence among young people most at risk in all French regions. After this research, more actions could be performed or suggested: offering an effective methodology for prevention and assistance in quitting smoking to French national education and presenting it to prevention and health promotion actors.

In conclusion, this trial aims to evaluate P2P, a promising peer-to-peer program for prevention against tobacco for young people in vocational high schools and is based on the TPB. It is specifically designed to examine the relationship between trial outcomes and fidelity of implementation in a goal of efficacy and replicability.

## Abbreviations

MDL: Maison des lycéens (student house)
TPB: Theory of Planned Behavior

## References

[1] Spilka S, Le Nézet O, Tovar M-L. Les drogues à 17 ans - premiers résultats de l’enquête ESCAPAD 2011. Tendances 2012;79.

[2] Anderson Johnson C, Palmer PH, Chou C-P, Pang Z, Zhou D, Dong L (2006). Tobacco use among youth and adults in mainland China: the China seven cities study. Public Health.

[3] Dappen A, Schwartz RH, O’Donnell RA (1996). Survey of adolescent smoking patterns. J AmBoard Family Practice.

[4] Kaminski A, Nauerth A, Pfefferle PI (2008). Health status and health behaviour of apprentices in the first year of apprenticeship - first results of a survey in vocational training schools in Bielefeld. Gesundheitswesen.

[5] Labalette C, Beetlestone E, Lert F. Impact de la loi Evin sur la consommation de tabac des lycéens. Résultats de l’enquête menée auprès de lycées professionnels d’Ile de France. Crips Ile-de-France; 2005 oct p. 36. http://fulltext.bdsp.ehesp.fr/Crips/Rapports/2005/T04016.pdf. Accessed 15 Jan 2013.

[6] Minary L, Martini H, Wirth N, Thouvenot F, Acouetey S, Maire C (2011). Tobacco use characteristics among apprentices in vocational centers. Rev Epid Sant Publique.

[7] Ruiz I, Ledesert B. Les comportements addictifs des jeunes de 15 à 24 ans. Montpellier: Observatoire Régional de la Santé du Languedoc Roussillon ORS LR. 2008. http://www.bdsp.ehesp.fr/Fulltext/391319. Accessed 15 dec 2012.

[8] Turner L, Mermelstein R, Flay B (2004). Individual and contextual influences on adolescent smoking. Ann N Y Acad Sci.

[9] Minary L (2011). Tabado : évaluation d’un programme d’aide au sevrage TABagique pour les ADOlescents en centres de formation des apprentis (CFA).

[10] Legleye S, Spilka S, Le Nézet O, Laffiteau C. Les drogues à 17 ans - résultats de l’enquête ESCAPAD 2008. Tendances OFDT. 2009;66:6.

[11] Bloch D, Chamonard D, Hocquaux C (2001). Les parcours scolaires et l’âge des bacheliers. Education et Formations.

[12] Lalonde M, Heneman B. La prévention du tabagisme chez les jeunes. Institut national de santé publique du Québec. 2004; http://www.inspq.qc.ca/pdf/publications/324-AvisPreventionTabagismeJeunes.pdf. Accessed 20 Mar 2013

[13] Conrad KM, Flay BR, Hill D (1992). Why children start smoking cigarettes: predictors of onset. Br J Addict.

[14] Peers KK (2003). Adolescent smoking. Addiction.

[15] Leventhal H, Cleary PD (1980). The smoking problem: a review of the research and theory in behavioral risk modification. Psychol Bull.

[16] Tyas SL, Pederson LL (1998). Psychosocial factors related to adolescent smoking: a critical review of the literature. Tob Control.

[17] Jacobson PD, Lantz PM, Warner KE, Wasserman J, Pollack HA, Ahlstrom AK. Combating teen smoking: research and policy strategies: The University of Michigan Press; 2001.

[18] Gouvernement du Canada SC. Enquête de surveillance de l’usage du tabac au Canada (ESUTC). 2002:2005. https://www.canada.ca/fr/sante-canada/services/publications/vie-saine/enquete-surveillance-usage-tabac-canada-esutc-2011.html. Accessed 20 Mar 2013

[19] Jensen Arnett J (2007). The myth of peer influence in adolescent smoking initiation. Health Educ Behav.

[20] Moan IS, Quitting Smoking RJ (2005). Applying an extended version of the theory of planned behavior to predict intention and behavior. J Appl Biobehav Res.

[21] Pageau M, Choinière R, Ferland M, Sauvageau Y (2001). Portrait de santé - Le Québec et ses régions. Édition 2001. Les publications du Québec.

[22] Lynch BS, Bonnie RJ. Growing up tobacco free. Preventing nicotine addiction in children and youths. Washington, D.C.: The National Academies Press; 1994. http://www.nap.edu/catalog.php?record_id=4757. Accessed 10 feb 2013. 25144107

[23] U.S. Department of Health and Human Service. Reducing Tobacco Use: A Report of the Surgeon General. Atlanta, Georgia: U.S: Department of Health and Human Services, Centers for Disease Control and Prevention, National Center for Chronic Disease Prevention and Health Promotion, Office on Smoking and Health; 2000 p. 474. http://www.cdc.gov/tobacco/data_statistics/sgr/2000/complete_report/index.htm. Accessed 10 feb 2013.

[24] Kim S, Crutchfield C, Williams C, Hepler N (1998). Toward a new paradigm in substance abuse and other problem behavior prevention for youth : youth development and empowerment approach. J Drug Educ.

[25] ONUDC/ Réseau mondial de la jeunesse. Pair à pair : utiliser les stratégies du pair à pair dans le domaine de la prévention de la toxicomanie. Nations Unies. New York: ONU; 2004 .http://www.unodc.org/pdf/youthnet/action/message/handbook_peer_frensh.pdf. Accessed 20 Mar 2013.

[26] Huteau M-E, Granier A, Arino A, Davy-Aubertin C, Bénézis C, Stoebner-Delbarre A (2012). « Sans clope, je suis au top ! » : quand l’éducation par les pairs fait un tabac. La Santé de. L’Homme.

[27] Huteau M-E, Granier A, Arino A, Davy-Aubertin C, Bénézis C, Stoebner-Delbarre A (2012). Sans clope, je suis au top ! : un projet de prévention communautaire. Revue de la santé scolaire et universitaire de l’AFPSSU.

[28] Le Grand E. « ESPAIR » Rapport d’évaluation. Fonds d’Expérimentation pour la Jeunesse; 2012. Report No.: AP2 – N°044–011–167-003. http://www.experimentation.jeunes.gouv.fr/IMG/pdf/Rapport_final_AP2_Evaluation_par_les_pairs.pdf. Accessed 10 feb 2013.

[29] Aveyard P, Cheng KK, Almond J, Sherratt E, Lancashire R, Lawrence T (1999). Cluster randomised controlled trial of expert system based on the transtheoretical (stages of change) model for smoking prevention and cessation in schools. BMJ.

[30] Cameron R, Brown KS, Best JA, Pelkman CL, Madill CL, Manske SR, Payne ME (1999). Effectiveness of a social influences smoking prevention program as a function of provider type, training method, and school risk. Am J Public Health.

[31] Peterson AV, Kealey KA, Mann SL, Marek PM, Sarason IG (2000). Hutchinson smoking prevention project : long-term randomized trial in school-based tobacco use prevention-results on smoking. J Natl Cancer Inst.

[32] Villet H, Lefebvre A, Frappier M (2007). Les « Suricates » - Projet de formation par les pairs dans le cadre de la prévention du tabagisme chez les jeunes : évaluation d’impact.

[33] Koumi I, Tsiantis J (2001). Smoking trends in adolescence: report on a Greek school-based, peer-led intervention aimed at prevention. Health Promot Int.

[34] Perry CL, Stigler MH, Arora M, Reddy KS (2009). Preventing tobacco use among young people in India: project MYTRI. Am J Public Health.

[35] Audrey S, Cordall K, Moore L, Cohen D, Campbell R (2004). The development and implementation of a peer-led intervention to prevent smoking among secondary school students using their established social networks. Health Educ J.

[36] Audrey S, Holliday J, Campbell R (2006). It’s Good to talk : adolescent perspectives of an informal, peer-led intervention to reduce smoking. Soc Sci Med.

[37] Campbell R, Starkey F, Holliday J, Audrey S, Bloor M, Parry-Langdon N (2008). An informal school-based peer-led intervention for smoking prevention in adolescence (ASSIST): a cluster randomised trial. Lancet.

[38] Starkey F, Audrey S, Holliday J, Moore L, Campbell R (2009). Identifying influential young people to undertake effective peer-led health promotion: the example of a stop smoking in schools trial (ASSIST). Health Educ Res.

[39] Azorin J-C, Burcheri L, Lhomost M (2012). Face à l’éducation par les pairs, quel positionnement pour les adultes référents ? La Santé de. L’Homme.

[40] Godin G, Kok G (1996). The theory of planned behavior: a review of its applications to health-related behaviors. Am J Health Promo.

[41] Schwenk G, Möser G (2009). Intention and behavior: a Bayesian meta-analysis with focus on the Ajzen–Fishbein model in the field of environmental behavior. Qual Quan.

[42] Topa G, Moriano JA. Theory of planned behavior and smoking: meta-analysis and SEM model. Subst Abuse Rehabil. 2010:23. 10.2147/SAR.S15168 PMC3819188 24474850

[43] Trafimow D, Sheeran P, Conner M, Finlay KA (2002). Evidence that perceived behavioural control is a multidimensional construct: perceived control and perceived difficulty. Br J Soc Psychol.

[44] McEachan RRC, Conner M, Taylor NJ, Lawton RJ (2011). Prospective prediction of health-related behaviours with the theory of planned behaviour: a meta-analysis. Health Psychol Rev.

[45] Moan IS (2005). Smoking or not smoking? How well does the theory of planned behaviour predict intention and behaviour? [Oslo]: Norwegian Institute for Alcohol and Drug Research.

[46] Conner M, Sandberg T, McMillan B, Higgins A (2006). Role of anticipated regret, intentions and intention stability in adolescent smoking initiation. Br J Of Health Psychol.

[47] Higgins A, Conner M (2003). Understanding adolescent smoking: the role of the theory of planned behaviour and implementation intentions. Psychol health med.

[48] McMillan B, Higgins AR, Conner M (2005). Using an extended theory of planned behaviour to understand smoking amongst schoolchildren. Addict Res Theory.

[49] Wiium N, Breivik K, Wold B (2006). The relationship between smoker role models and intentions to smoke among adolescents. J Youth Adolesc.

[50] Webb TL, Sniehotta FF, Michie S (2010). Using theories of behaviour change to inform interventions for addictive behaviours. Addiction.

[51] Michie S, Abraham C (2008). Advancing the science of behaviour change: a plea for scientific reporting. Addiction.

[52] Hardeman W, Johnston M, Johnston DW, Bonetti D, Wareham NJ, Kinmonth AL (2002). Application of the theory of planned behaviour in behaviour change interventions: a systematic review. Psychol Health.

[53] Quine L, Rutter DR, Arnold L (1998). Predicting and understanding safety helmet use among schoolboy cyclists: a comparison of the theory of planned behaviour and the health belief model. Psychol Health.

[54] Ajzen I (1991). The theory of planned behavior. Organ Behav Hum Decis Process.

[55] Webb TL, Joseph J, Yardley L, Michie S (2010). Using the internet to promote health behavior change: a systematic review and meta-analysis of the impact of theoretical basis, use of behavior change techniques, and mode of delivery on efficacy. J Med Internet Res.

[56] Cooke R, French DP (2008). How well do the theory of reasoned action and theory of planned behaviour predict intentions and attendance at screening programmes? A meta-analysis. Psychol Health.

[57] Albarracín D, Johnson BT, Fishbein M, Muellerleile PA (2001). Theories of reasoned action and planned behavior as models of condom use: a meta-analysis. Psychol Bull.

[58] Armitage CJ, Conner M (2000). Social cognition models and health behaviour: a structured review. Psychol Health.

[59] Schulze R, Wittmann WW. A meta-analysis of the theory of reasoned action and the theory of planned behavior: the principle of compatibility and multidimensionality of beliefs as moderators. In: Schulze R, Holling H, Böhning D, éditeurs. Meta-analysis: new developments and applications in medical and social sciences. Ashland: Hogrefe & Huber Publishers; 2003. p. 219–250.

[60] Armitage CJ, Conner M (2001). Efficacy of the theory of planned behaviour: a meta-analytic review. Br J Soc Psycho.

[61] Hagger MS, Chatzisarantis NLD, Biddle SJH (2002). A meta-analytic review of the theories of reasoned action and planned behavior in physical activity: predictive validity and the contribution of additional variables. J Sport Exerc Psychol.

[62] Bazillier C. Etude longitudinale des effets de facteurs psychosociaux et d’interventions de prévention sur la susceptibilité de fumer d’enfants de huit à onze ans : le cas de la Théorie du Comportement Planifié et du Réseau Amical. Paris 10; 2009. http://www.theses.fr/2009PA100153. Accessed 21 Mar 2013.

[63] Kothe EJ, Mullan BA, Butow P (2012). Promoting fruit and vegetable consumption. Testing an intervention based on the theory of planned behaviour. Appetite.

[64] Elliott MA, Armitage CJ (2009). Promoting drivers’ compliance with speed limits: testing an intervention based on the theory of planned behaviour. Br J Psychol.

[65] Spilka S, Le Nézet O, Tovar ML. Estimation 2011 des consommations de produits psychoactifs à 17 ans. Saint-Denis: Observatoire Français des Drogues et des Toxicomanies OFDT; 2011. http://www.ofdt.fr/BDD/publications/docs/eisxstra.pdf. Accessed 20 Mar 2013.

[66] Ajzen I. Constructing a Theory of Planned Behavior Questionnaire. http://people.umass.edu/aizen/pdf/tpb.measurement.pdf. Accessed 22 Mar 2013.

[67] Stoebner-Delbarre A, Djoufelkit K, Nguyen C, Adam de Villiers M, Bousquet M, Lemonnier G (2007). Enquête DAST décembre 2006 «Devenir Académie sans Tabac» Lycées sans tabac en Languedoc-Roussillon. Epidaure.

[68] Stoebner-Delbarre A, Djoufelkit K, Nguyen C, Adam de Villiers M, Bousquet M, Lemonnier G. Enquête DAST décembre 2007 «Devenir Académie sans Tabac» Lycées sans tabac en Languedoc-Roussillon Epidaure; 2008 https://bdoc.ofdt.fr/index.php?lvl=notice_display&id=14565 Acessed 15 Mar 2013.

[69] Stoebner-Delbarre A, Nguyen C. Projet Arrêt tabac jeunes libres comme L’R. Rapport Epidaure. Epidaure; 2004 juill.

[70] Stoebner-Delbarre A, Nguyen C, Couralet D. Action 5+1 : Smoking cessation program for youth. Caen; 2004.

[71] DiFranza JR, Savageau JA, Rigotti NA, Fletcher K, Ockene JK, McNeill AD (2002). Development of symptoms of tobacco dependence in youths: 30 month follow up data from the DANDY study. Tob Control.

[72] Etude NICO / NDIT Study. http://ndit.crchum.qc.ca/main.php?p=6&lang=fr. Accessed 22 Mar 2013.

[73] Beck F, Guignard R, Richard JB. Les niveaux d’usage des drogues en France en 2010 - Exploitation des données du Baromètre santé. Tendances OFDT. juin 2011 http://www.bdsp.ehesp.fr/Fulltext/436431/. Accessed 20 Mar 2013.

[74] Spilka S, Le Nézet O, Beck F, Ehlinger V, Godeau E. Alcool, tabac et cannabis durant les « années collège ». Tendances OFDT 2012 https://www.ofdt.fr/publications/collections/periodiques/lettre-tendances/alcool-tabac-cannabis-durant-annees-college-tendances-80-avril-2012/. Accessed 20 Mar 2013.

[75] Verbeke G, Molenberghs G, Thijs H, Lesaffre E, Kenward MG (2000). Sensitivity analysis for non-random dropout: a local influence approach. Biometrics.

[76] Hawe P, Shiell A, Riley T, Gold L (2004). Methods for exploring implementation variation and local context within a cluster randomised community intervention trial. J Epidemiol Community Health.

[77] Dagnaud M, Génération Y. Les jeunes et les réseaux sociaux, de la dérision à la subversion: Les Presses de Sciences Po; 2011.

